# Impact of Indoor Air Pollution on Occupational Exposure: Rethinking the Major Health Threat for Airport Workers

**DOI:** 10.3390/life16081242

**Published:** 2026-07-27

**Authors:** Alessia Perna, Adriano Paolucci, Teresa Esposito, Giulia Spernanzoni, Annalisa Bruno, Rosa Maria Russo, Maria Pierdomenico, Massimo Santoro

**Affiliations:** 1 USMAF-SASN Lazio Umbria Marche Sardegna, Via Mario Stoppani, 20, 00054 Rome, Italy; t.esposito@sanita.it (T.E.); g.spernanzoni@sanita.it (G.S.); a.bruno@sanita.it (A.B.); rm.russo@sanita.it (R.M.R.); 2 Electronics Engineering for Automation and Telecommunication, University of Sannio, Corso Garibaldi 107, 82100 Benevento, Italy; a.paolucci-esterno@sanita.it; 3 Division of Biotechnologies, Italian National Agency for New Technologies, Energy and Sustainable Development (ENEA), 00123 Rome, Italy; maria.pierdomenico@enea.it (M.P.); massimo.santoro@enea.it (M.S.)

**Keywords:** occupational exposure, airport workers, indoor pollution, ultrafine particles, biomarkers, health

## Abstract

**(1) Background:** Airports workers are exposed to a mixture of airborne pollutants generated by aircraft operations, ground support equipment, road traffic, and indoor microenvironments. Indoor air quality, influenced by outdoor pollutant and ventilation dynamics, represents an important, but overlooked determinant of occupational exposure. Fine and ultrafine particulate matter (PM) can induce oxidative stress, inflammation, and xenobiotic responses, affecting not only the respiratory system, but also other organs. **(2) Methods:** This review examines the health effects of occupational exposure among airport workers, with emphasis on indoor air pollution besides aircraft engine emissions. Studies addressing exposure characterization, biological effects, biomarkers, and risk management strategies were critically evaluated. **(3) Results:** Occupational exposure is driven by both combustion-derived pollutants and indoor–outdoor air exchange processes. Ultrafine particles, black carbon, polycyclic aromatic hydrocarbons, and trace metals contribute to oxidative stress, inflammatory responses, and xenobiotic pathway activation. Monitoring indoor microclimatic parameters, including temperature, atmospheric pressure, and humidity, may facilitate the identification of event-related deterioration in indoor air quality. **(4) Conclusions:** Indoor air pollution should be recognized as a key component of airport occupational exposure. Integrating indoor/outdoor air quality monitoring and biomarker-based surveillance may improve risk assessment and support more effective protection of airport workers, while advanced predictive tools, including AI-based exposure modelling, represent a promising direction that still requires dedicated validation.

## 1. Introduction

Air pollution in urban environments has been consistently associated with an increased risk of cardiovascular and respiratory diseases, as well as cancer development. Particular attention has been focused on ultrafine particles (UFPs; aerodynamic diameter ≤ 100 nm), which can penetrate deep into the respiratory tract, deposit in the alveolar region, and, owing to their small size, translocate into the bloodstream. Consequently, indoor air pollution represents a major public health concern, as exposure to fine and ultrafine particulate matter can induce oxidative stress, inflammation, and xenobiotic responses, affecting not only the respiratory system but also other organs and tissues. Recent in vitro studies have demonstrated that even low-dose exposure to indoor fine and ultrafine particulate matter can induce gene expression changes in bronchial epithelial cells, including upregulation of the aryl hydrocarbon receptor (*AhR*) and xenobiotic metabolism genes (*CYP1A1* and *CYP1B1*), without corresponding changes in DNA promoter methylation, suggesting the involvement of alternative epigenetic mechanisms [1].

In this context, airports—both military and commercial, regardless of their size—have emerged as important sources of atmospheric emissions and have become a focus of research on air quality and related health effects. Such concerns involve not only passengers but also airport workers, who may experience prolonged occupational exposure to airborne pollutants. Although conventional air quality models are effective in estimating pollutant concentrations over large spatial scales, their application in airport environments remains challenging because workers are exposed not only to emissions from aircraft engines but also to a variety of indoor and workplace-related pollution sources.

Comprehensive aerosol characterization includes the determination of gravimetric mass concentrations of particulate matter (PM_10_, PM_2.5_ and PM_1_), particle number size distributions, and black carbon (BC) levels. Human exposure to aircraft-related emissions is influenced by multiple factors, including airport location and infrastructure, aircraft engine and fuel characteristics, fuel carbon content, and the activity of ground support vehicles. In addition, environmental processes affecting pollutant dispersion and transformation play a significant role. These processes are influenced by the composition of volatile and non-volatile compounds, meteorological conditions (e.g., wind speed and direction, temperature, and relative humidity), seasonal and weekly variability, and the contribution of other emission sources in the surrounding area, particularly road traffic.

Within this framework, monitoring indoor microclimatic parameters, including temperature, atmospheric pressure, and relative humidity, can facilitate the identification of event-related deterioration in indoor air quality and the assessment of the effects of routine activities, such as opening or closing doors and windows.

Furthermore, temperature and relative humidity regulated by heating, ventilation, and air conditioning (HVAC) systems can influence indoor aerosol dynamics by affecting particle hygroscopic growth, condensation processes, and the partitioning of semi-volatile compounds between the gas and particle phases. These processes may modify particle size distribution, atmospheric persistence, and the potential toxicological properties of indoor aerosols, thereby influencing human exposure assessment [2,3].

Despite the growing body of evidence on airport-related air pollution, data specifically addressing the relationship between occupational exposure profiles and indoor air quality among airport workers remain limited. A better understanding of this relationship is essential for identifying exposure determinants and minimizing potential health risks. Therefore, the aim of this mini-review is to investigate the potential adverse health effects associated with occupational exposure to airport-related indoor air pollution, identify biomarkers that may be useful for exposure and health monitoring, and evaluate risk management strategies to protect airport workers.

## 2. Materials and Methods

To the best of our knowledge, a limited number of studies have investigated the potential adverse health effects, particularly respiratory outcomes, associated with occupational exposure to indoor air pollution among civil airport workers. Therefore, this study was designed as a structured mini-narrative review aimed at summarizing the current evidence on occupational exposure to indoor air pollution in airport environments, with particular emphasis on particulate matter (PM), ultrafine particles (UFPs), biomarkers of exposure and biological effects, and potential risk management strategies. A structured literature search was performed using the PubMed, Scopus, and Web of Science databases. The search covered publications available from January 1999 to May 2026 and included combinations of the following keywords: airport workers, indoor air pollution, particulate matter, ultrafine particles, aircraft emissions, health effects, biomarkers, oxidative stress, inflammation, occupational exposure, and risk assessment. The search identified 35 records, of which 29 studies met the eligibility criteria and were included in this mini-narrative review. Studies were selected based on their relevance to occupational exposure to indoor air pollution in civil airport settings and their evaluation of health effects among airport workers. Eligible studies included investigations of exposure characterization, respiratory and systemic health outcomes, biomarkers of exposure or biological effects, and occupational risk assessment. Studies focusing exclusively on outdoor environmental monitoring without relevance to indoor occupational exposure or passenger exposure, or non-airport occupational settings were excluded.

### Search Strategy and Study Selection

**Figure 1:**
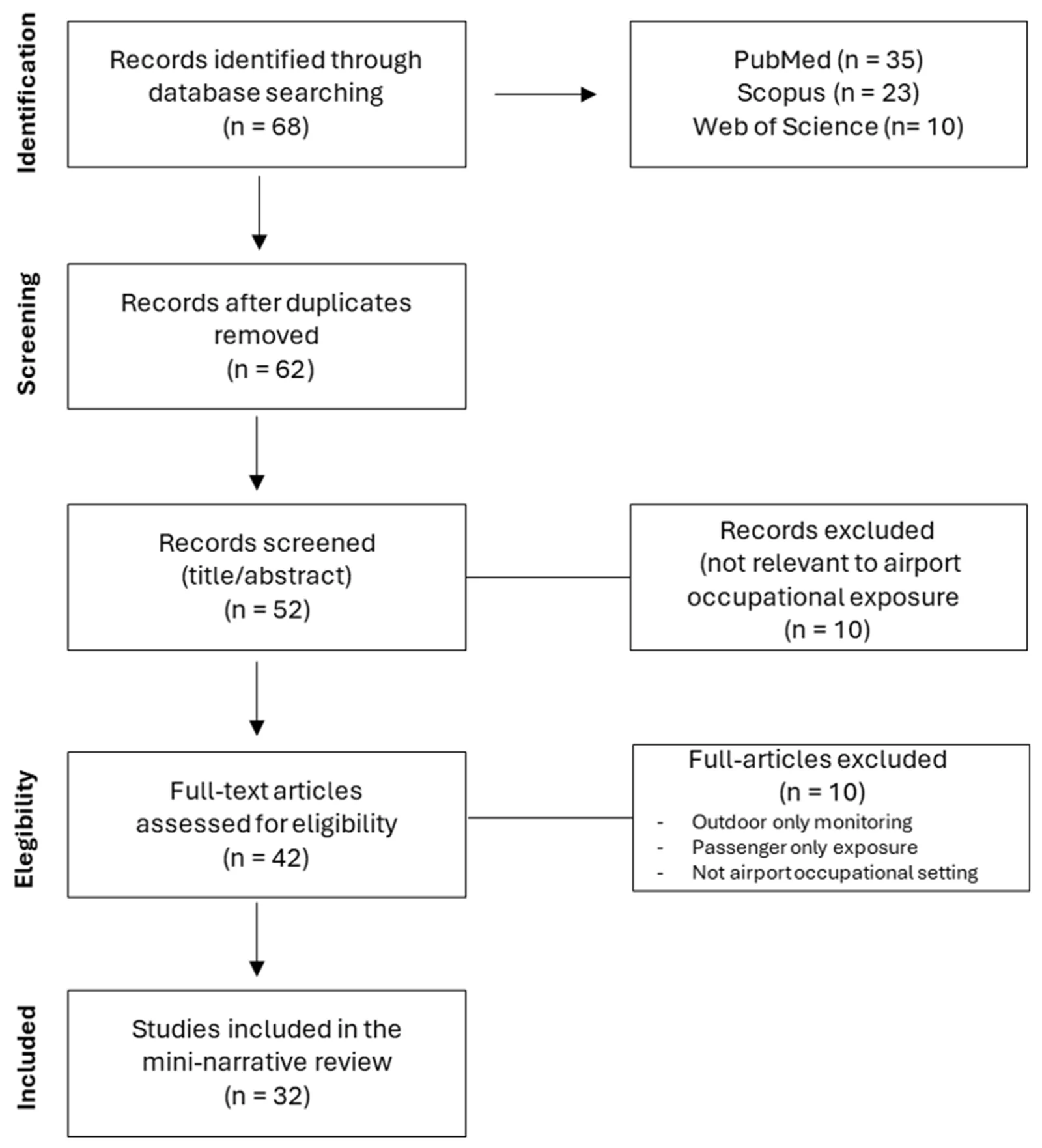
Flow diagram of the identification, screening and selection of studies included in this mini-narrative review.

The literature search and study selection process is summarized in Figure 1. Records identified across the three databases were first de-duplicated and screened at the title/abstract level; the full text of potentially eligible records was then retrieved and assessed against the inclusion and exclusion criteria described above (Table 1). This structured, PRISMA-informed selection process, rather than a single-database search, was adopted to reduce the risk of missing relevant environmental, occupational, and engineering literature.

## 3. Results

### 3.1. Sources and Formation of Airborne Pollutants in Airport Occupational Environments

Airport workers are exposed to airborne pollutants originating from multiple, spatially overlapping sources. Aircraft engines and auxiliary power units, diesel-powered ground support equipment (baggage tractors, high-loaders, pushback tugs), and landside road traffic all act as combustion sources that directly emit primary pollutants, including ultrafine particles (UFPs), black carbon, polycyclic aromatic hydrocarbons (PAHs), trace metals, and gaseous co-pollutants such as nitrogen oxides and volatile organic compounds. In addition to these primary emissions, secondary aerosol can form in the airport atmosphere through photochemical reactions and through condensation and gas-to-particle partitioning of semi-volatile compounds, processes that are themselves modulated by meteorological conditions (temperature, humidity, wind speed and direction) and by HVAC-driven microclimatic changes indoors, as introduced in Section 1.

**Figure 2:**
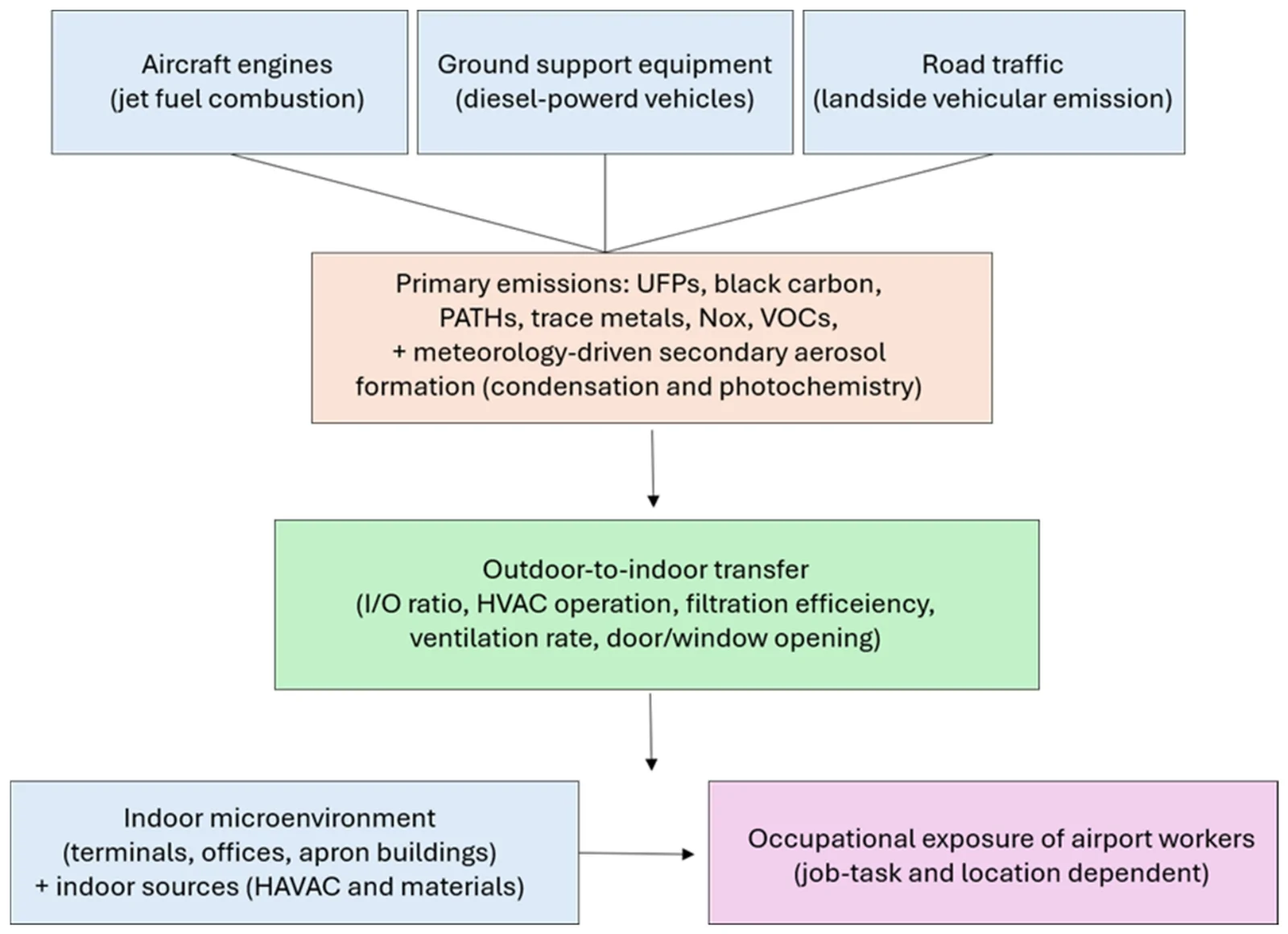
Schematic overview of outdoor combustion sources, atmospheric transformation, and outdoor-to-indoor transfer processes contributing to occupational exposure in airport environments.

These outdoor-origin pollutants are not confined to open-air areas: they infiltrate indoor microenvironments (terminals, offices, apron-facing buildings) through door- and window-opening events and through HVAC systems, with the extent of infiltration governed by indoor/outdoor (I/O) ratios, ventilation rate, and filtration efficiency. Indoor air quality therefore results from the combination of this outdoor-to-indoor transfer with genuinely indoor sources (e.g., HVAC operation, building materials, cleaning activities), so that occupational exposure of airport workers is shaped jointly by outdoor emission intensity, indoor/outdoor exchange dynamics, and job-specific proximity to combustion sources. This overall framework, summarized schematically in Table 2 and Figure 2, underlies the exposure patterns described in the remainder of this section.

### 3.2. Occupational Exposure Among Airport Workers

Most of the studies included in this review primarily investigated the adverse respiratory health effects associated with occupational exposure to aircraft engine emissions and jet fuel combustion products. These emissions contain a complex mixture of pollutants, including nitrogen oxides (NO_x_), ozone (O_3_), sulfur dioxide (SO_2_), and particulate matter (PM), which are comparable in several respects to pollutants found in motor vehicle exhaust. Many of the investigated populations consisted of male airport workers employed in operational and ground-handling activities.

One of the earliest studies addressing occupational exposure among airport workers was conducted by Tunnicliffe et al. in 1999 [4]. The authors classified workers into three exposure categories according to their job tasks and the estimated intensity of exposure to airport-related airborne pollutants. This classification enabled the evaluation of potential associations between occupational exposure and respiratory health outcomes:

1. **High-exposure group**: workers directly involved in aircraft servicing operations, including baggage handlers, airport ground staff, marshallers, operational engineers, fitters, and engineering technicians.
2. **Medium-exposure group**: workers operating on the apron, inside aircraft, or within terminal areas, including security personnel, firefighters, and airfield operations managers.
3. **Low-exposure group**: administrative personnel, terminal employees, and office workers.

This classification allowed for comparison of occupational exposure levels according to job tasks and working locations. Exposure levels were higher among workers involved in activities closer to aircraft operations compared with administrative and office personnel. These findings suggest that occupational exposure among airport workers is largely determined by job-specific tasks, work location, and fluctuations in aircraft and ground-support operations.

Subsequently, Yang et al. [5] investigated whether the prevalence of acute and chronic respiratory symptoms differed between 106 airport workers (exposed group) and 305 terminal or office workers (control group) at Kaohsiung International Airport (KIA), Taiwan. The authors reported a significantly higher prevalence of chronic respiratory symptoms among exposed workers, particularly cough and dyspnea; after adjustment for confounders, the adjusted ORs were all greater than the corresponding crude ORs (Table 3).

In addition to epidemiological investigations, numerous studies have focused on the characterization of aircraft-related emissions and air quality at major international airports worldwide. Emission monitoring and aerosol measurement campaigns have been conducted at several airports, including Amsterdam Airport Schiphol (AMS) [6], Rome Ciampino Airport (CIA) [7], London Heathrow Airport (LHR) [8], Beirut–Rafic Hariri International Airport (RHIA) [9], Hartsfield–Jackson Atlanta International Airport [10], Los Angeles International Airport (LAX), and other major airports in California [11]. These studies consistently reported elevated concentrations of ultrafine particles, black carbon, and combustion-related pollutants in areas located near aircraft operations.

Overall, the included studies reported higher airborne pollutant concentrations among workers involved in ground-handling and aircraft servicing activities compared with administrative and office personnel. Exposure levels varied according to job tasks, distance from aircraft operations, airport traffic conditions, and investigated environmental parameters. Several studies [12,13,14] have demonstrated that aircraft engines represent a major source of air pollutants within airport environments and in areas located near aircraft operations. The principal pollutants emitted by jet engines include nitrogen oxides (NO_x_), carbon dioxide (CO_2_), carbon monoxide (CO), volatile organic compounds (VOCs) (including low-molecular-weight polycyclic aromatic hydrocarbons (PAHs)), sulfur dioxide (SO_2_), particulate matter (PM), ultrafine particles (UFPs; typically ranging from 23 to 36 nm in diameter), and trace metals. These pollutants are generated during fuel combustion and contribute significantly to the deterioration of air quality in airport occupational settings.

Among these emissions, UFPs are of particular concern because of their ability to penetrate deep into the respiratory tract, reach the alveolar region, and potentially enter the systemic circulation. Several studies on occupational exposure to UFPs among workers operating near aircraft emission showed an association with adverse respiratory and cardiovascular health effects [15].

### 3.3. Ultrafine Particles (UFPs)

More recently, the Danish Centre for Environment and Energy [16] reported elevated concentrations of ultrafine particles (UFPs) at Copenhagen Airport (CPH), primarily associated with jet fuel combustion and diesel-powered ground support equipment. These concentrations were particularly high in apron areas and exceeded levels typically observed in urban background environments. Emission characterization studies conducted with an Italian Air Force jet equipped with a Rolls-Royce Spey engine confirm that both fossil fuel and biofuel blend combustion generate ultrafine particles predominantly below 300 nm, with biofuel blends emitting a higher number of nanoparticles (up to 2.6-fold increase compared to fossil fuel), particularly in the sub-40 nm range. These particles, with their high surface area and capacity to carry adsorbed toxicants, represent a significant inhalation hazard for ground personnel working near taxiing or idling aircraft [17].

In a study by Møller et al. [18], individual exposure profiles to UFPs were assessed among five occupational groups at CPH, including baggage handlers, catering drivers, cleaning staff, airside security personnel, and landside security personnel. A total of 30 employees were equipped with a portable monitoring system combining a GPS device and a NanoTracer, enabling real-time measurements of particle number concentration and size distribution (approximately 10–300 nm). Data were collected over eight working days in October 2012, between 07:30 and 15:00.

The study demonstrated a clear exposure gradient to UFPs across occupational categories. Consistent with previous findings (e.g., Tunnicliffe et al. and other earlier studies), workers could be stratified into three exposure groups: (i) a high-exposure group comprising baggage handlers; (ii) a medium-exposure group including catering drivers, cleaning staff, and airside security personnel; and (iii) a low-exposure group consisting of landside security personnel (Table 4). These results showed differences in UFP exposure intensity among occupational categories, with higher exposure levels observed in workers operating closer to aircraft-related activities.

Overall, this study demonstrated a clear occupational exposure gradient to ultrafine particles (UFPs), with the highest concentrations measured in the apron area. Baggage handlers were exposed to average concentrations approximately seven times higher than those recorded for indoor employees. The integration of personal monitoring and GPS tracking allowed for the assessment of spatial and temporal variability in UFP exposure among different occupational categories. However, exposure assessment was limited to daytime working hours and a single season (autumn), highlighting the need for longitudinal measurements to better define comprehensive indoor–outdoor exposure profiles. Supporting these findings, a study conducted in an urban university classroom in Rome demonstrated that indoor UFPs, particularly traffic-related nanoparticles carrying polycyclic aromatic hydrocarbons (PAHs), can penetrate indoor environments and trigger xenobiotic responses in human bronchial epithelial cells exposed at the air-liquid interface, even at PM_2.5_ mass concentrations well below WHO (World Health Organization) guidelines [19]. These results underscore the role of indoor UFPs as carriers of toxic molecules, with their biological impact being modulated by meteorological conditions such as rainfall and wind speed, factors equally relevant in airport microenvironments.

Consistent with these findings, Touri et al. [14] reported that particulate matter concentrations are inversely related to the distance from airport operational areas, with progressively lower pollutant levels observed as distance from the airport platform increases. These results reinforce the strong spatial dependence of airport-related air pollution.

Jet fuel, with kerosene as its primary component, represents the main source of aircraft emissions. Aircraft exhaust is dominated by ultrafine particles, typically smaller than 20 nm. In addition, measurements conducted near Los Angeles International Airport (LAX; Los Angeles, CA, USA) have identified aircraft-derived UFPs with size distributions ranging approximately from 11 nm to 80–90 nm in diameter, further confirming the predominance of nanoscale particulate emissions in airport environments [14].

### 3.4. Polycyclic Aromatic Hydrocarbons (PAHs)

Interestingly, exposure to toxic compounds via contaminated bleed air from engine compressors has been widely investigated in cabin crews and pilots. This exposure pathway may involve organophosphate esters (OPEs), which have been associated with adverse neurological outcomes and respiratory symptoms, suggesting potential systemic effects beyond the respiratory tract. In vitro direct exposure studies using BEAS-2B bronchial epithelial cells at the air-liquid interface have further shown that both fossil jet fuel and biofuel blend emissions induce acute oxidative stress responses, evidenced by upregulation of the heme oxygenase-1 (HO-1) gene, a sensitive biomarker of oxidative damage in lung tissue [17]. Notably, biofuel blends elicited a lower immediate response than fossil fuel, although a recovery period of one-hour post-exposure was sufficient to dramatically increase HO-1 expression, highlighting the acute and time-dependent nature of the oxidative burst triggered by aviation emissions.

In addition, airport-related emissions contain a complex mixture of trace metals that may serve as chemical markers of aviation and ground-support activities [13]. These include molybdenum (Mo), calcium (Ca), sodium (Na), iron (Fe), copper (Cu), barium (Ba), chromium (Cr), aluminum (Al), silicon (Si), magnesium (Mg), cobalt (Co), manganese (Mn), vanadium (V), nickel (Ni), lead (Pb), titanium (Ti), and zirconium (Zr). The presence and relative abundance of these metals reflect multiple emission sources, including aircraft engines, fuel combustion processes, wear of mechanical components, and ground handling operations, and they contribute to the overall toxicological burden of airport air pollution. Polycyclic aromatic hydrocarbons (PAHs), including several known carcinogenic compounds, are also considered relevant chemical tracers of airport-related emissions. At Rome Fiumicino Airport (FCO), higher PAH concentrations have been reported in apron areas (27.2 µg/m^3^) compared with indoor environments such as airport buildings and passenger terminals, suggesting a strong influence of aircraft and ground-support activities on spatial exposure patterns [20].

In a study published in 2003 [21], Whelan et al. reported a higher prevalence of respiratory disorders among flight attendants compared with other female workers in the United States. The study combined real-time monitoring of PAH concentrations using a photoelectric aerosol sensor with integrated chemical analysis of airborne samples collected via low-volume air samplers. These samples were subsequently analysed by gas chromatography–mass spectrometry (GC–MS). The results consistently indicated elevated PAH levels across occupational settings associated with aviation activities, supporting the hypothesis of increased exposure in aircraft-related work environments.

Similarly, at Charles de Gaulle Airport (Paris, France) [22], occupational exposure assessments were conducted using individual portable monitoring devices, including carbon monoxide analysers and pumps for aromatic hydrocarbon sampling, that were worn by airport ground staff. These measurements provided additional evidence of measurable exposure to combustion-related pollutants in airport occupational settings and highlighted the relevance of personal monitoring approaches for exposure characterization.

Subsequent studies have not only focused on the identification of indoor air pollutants in airport environments but have also investigated the associated biological effects in exposed workers. In this context, Italian researchers evaluated the relationship between 23 individual polycyclic aromatic hydrocarbons (PAHs) and urinary levels of 1-hydroxypyrene, a widely used biomarker of PAH exposure, among workers at Fiumicino–Leonardo da Vinci Airport (Rome, Italy). In addition, genotoxic effects were assessed using micronucleus and comet assays performed on exfoliated buccal cells [12].

Higher concentrations of PAHs, predominantly methylnaphthalene and acenaphthylene, were detected in apron areas compared with indoor environments such as airport buildings and departure terminals. However, urinary 1-hydroxypyrene levels did not differ significantly between exposed workers and the control group. In contrast, the comet assay revealed evidence of DNA damage associated with oxidative stress, suggesting that exposure to airport-related pollutants may induce early biological effects even in the absence of clear differences in conventional exposure biomarkers [23,24].

Furthermore, in vitro studies conducted by a research group in Marseille demonstrated that ultrafine particles (UFPs) can modulate cytokine production, thereby influencing inflammatory pathways in human cells. These findings support a potential mechanistic link between exposure to airport-derived particulate pollution and inflammatory responses relevant to human health [25].

### 3.5. Biomarkers of Exposure to Indoor Pollutants

Subsequently, the toxicity of jet fuel has been investigated through biomarker-based approaches, with particular attention to the uptake of JP-8 components. Exposure to these compounds may occur via both inhalation and dermal contact. In this context, benzene and naphthalene, measured in air and in exhaled breath condensate (EBC), have been proposed as potential biomarkers of occupational exposure to jet fuel-related emissions [24].

Marie-Desvergne et al. [26] conducted a large study involving 458 airport workers from Marseille Provence Airport and Paris Charles de Gaulle Airport. Participants included personnel working directly on the apron (high-exposure group) and office-based staff (lower-exposure group). Exhaled breath condensate samples were collected to assess internal exposure, while ambient nanoparticle exposure was characterized in terms of particle number concentration, size distribution, and electron microscopy analysis.

The study demonstrated that airport workers experienced significantly higher particle number concentrations (1.0 × 10^4^–2.1 × 10^7^ particles/cm^3^) compared with office workers (10^3^–10^4^ particles/cm^3^, consistent with background levels influenced by traffic emissions). However, office workers were intermittently exposed to short-term peaks of 10^4^–10^5^ particles/cm^3^ when building doors were opened. In addition, exposed airport workers were subjected to significantly smaller particles (mean geometric diameter: 17.7 nm) compared with office workers (23.7 nm), indicating a shift toward ultrafine particle exposure in occupational settings with direct apron activity [27].

The impact of airliner-generated particles infiltrating indoor environments through open doors and heating, ventilation, and air-conditioning (HVAC) systems has also been investigated. In particular, the contribution of outdoor emissions to indoor air quality in airport terminals has been assessed by measuring fine particulate matter (PM_2.5_) concentrations across different seasons [28].

**Figure 3:**
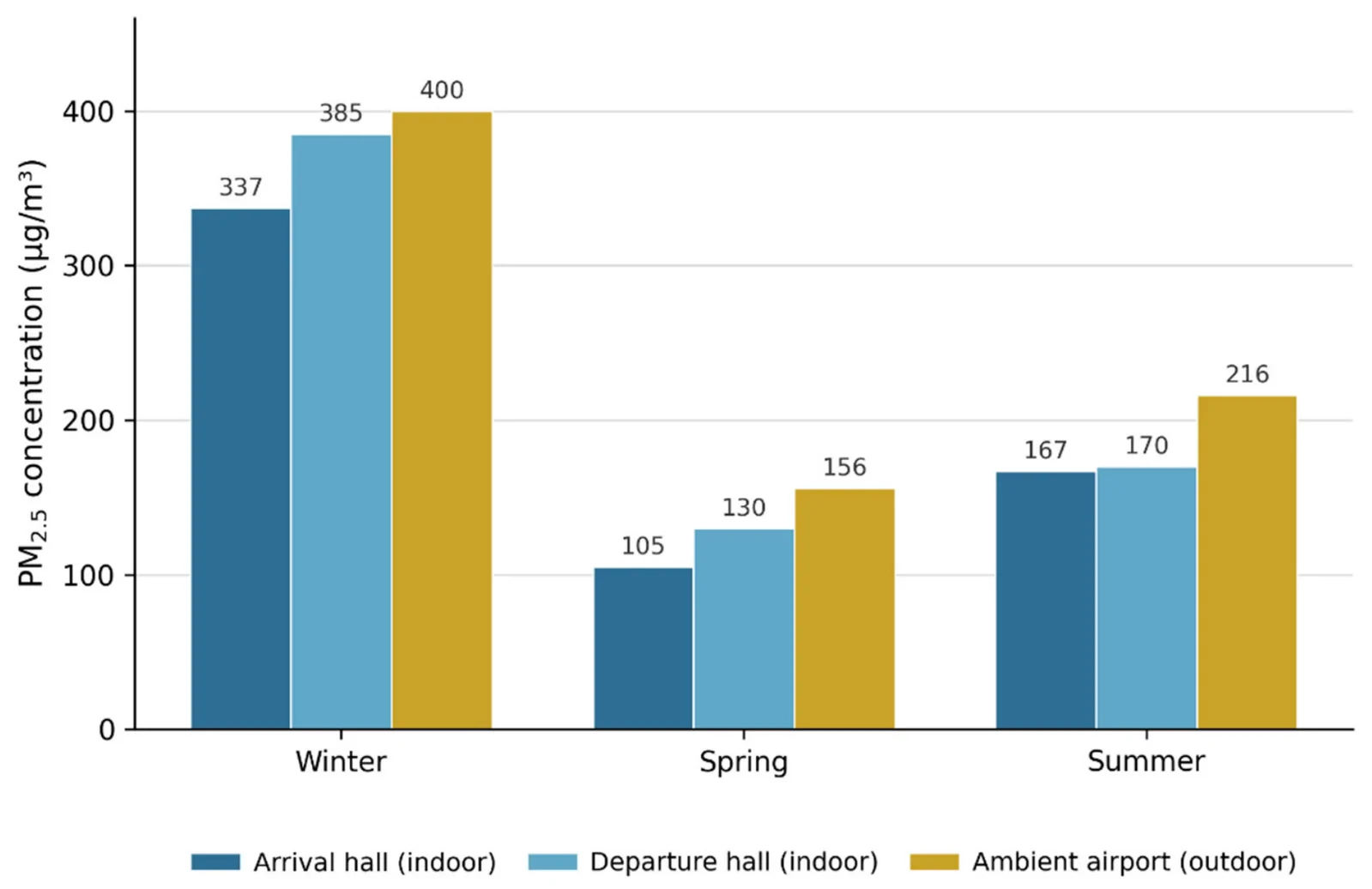
Seasonal indoor and outdoor PM_2.5_ concentrations at airport terminals. Values shown for the arrival hall and departure hall (indoor) and for ambient airport air (outdoor) across the three sampled seasons [29]. Black carbon and PAHs are not shown because the studies reviewed here did not report comparable paired indoor/outdoor or seasonal values for these pollutants: black carbon was only described qualitatively (elevated near combustion sources), and PAHs were only analyzed in a single apron-versus-indoor comparison (27.2 µg/m^3^ in apron areas) [20].

PM_2.5_ concentrations showed seasonal variability across indoor and outdoor airport environments (Figure 3). The highest concentrations were observed during winter in both terminal areas and ambient airport air, whereas lower concentrations were reported during spring and intermediate values during summer [29]. The comparison between indoor and outdoor PM_2.5_ concentrations provides an indication of the contribution of outdoor-origin particles to indoor airport air quality. Although specific indoor/outdoor (I/O) ratios differed among the investigated environments, the available evidence suggests that particle infiltration through ventilation systems, air exchange processes, and building operation conditions represents an important determinant of indoor PM_2.5_ levels [30]. HVAC systems may therefore play a dual role by reducing particle penetration through filtration while simultaneously influencing indoor particle transport depending on ventilation settings and operational characteristics.

## 4. Discussion

This mini-narrative review highlights that occupational exposure to indoor air pollution represents a relevant concern for airport workers, particularly due to the complex interaction between outdoor-origin emissions, indoor environmental conditions, and prolonged occupational exposure. The available evidence indicates that particulate matter, ultrafine particles, and combustion-derived pollutants represent key components of airport-related air pollution and may contribute to respiratory and systemic effects through mechanisms involving oxidative stress, inflammation, and biological responses measurable through specific biomarkers.

To date, indoor air pollution affecting aircraft and airport personnel has not yet been integrated into the International Civil Aviation Organization (ICAO) database. Furthermore, assessments evaluating the impact of pollutant emissions from aircraft engine exhaust on air quality typically focus exclusively on regional contributions and the immediate vicinity of airports, thereby neglecting the indoor environment. Consequently, indoor emissions have been systematically overlooked in airport inventories, and there are currently no comprehensive regulatory limits or guidelines to account for these specific air pollutants. Although prior studies [12,13,14] mapped air quality patterns near airports, they frequently overlooked population health outcomes and long-range chemical reactions. Traditional grid-based air quality models can effectively simulate pollutant concentrations across extensive areas; however, they present practical challenges when applied specifically to airport microenvironments. The evidence summarized in this review indicates that occupational exposure levels vary according to job tasks, working locations, and proximity to aircraft-related emission sources. Workers involved in aircraft servicing and ground-handling activities generally experience higher exposure levels compared with administrative personnel, reflecting the spatial distribution of emission sources and the characteristics of airport operations.

The present study addressed this knowledge gap by evaluating not only aircraft particle emission data gathered from international airports, but also exposure levels, temporal rhythms, and the confounding roles of other environmental factors. Indeed, indoor exposure patterns and subsequent biological responses are shaped not only by indoor sources and airflow regimes, but also by the infiltration of outdoor ambient pollution into the indoor environment, particularly fine and combustion-derived particles [31]. The seasonal variability observed in indoor PM_2.5_ concentrations suggests that outdoor-origin particles may substantially contribute to indoor airport air quality. This process is likely influenced by air exchange dynamics, ventilation characteristics, HVAC operation, filtration efficiency, and passenger-related activities such as door-opening events. Therefore, indoor airport environments should be considered dynamic systems where outdoor emissions and indoor conditions interact continuously.

Particulate matter (PM), a complex mixture of solid particles and liquid droplets, originates from both natural sources (such as crustal dust, pollen, and marine salts) and anthropogenic activities (including vehicular emissions and industrial processes). PM frequently acts as a carrier for xenobiotic toxicants, including heavy metals and organic pollutants, which adsorb onto its surface. Due to its aerodynamic diameter and chemical composition, PM can penetrate deeply into the respiratory tract, functioning as a physical vehicle that facilitates the systemic distribution and bio-accessibility of toxic substances to extrapulmonary organs [31,32].

Among airport-related pollutants, UFPs deserve particular attention because of their small aerodynamic diameter, high surface area, and potential to reach deeper regions of the respiratory system. The elevated UFP concentrations reported near aircraft operational areas suggest that workers performing ground activities may represent a population with increased exposure to combustion-derived nanoparticles.

Exposure to incomplete combustion products, including particulate matter (PM), benzene, benzo[a]pyrene, and carbon monoxide derived from biomass fuels is associated with a wide range of adverse health outcomes. These include respiratory diseases such as asthma and chronic obstructive pulmonary disease (COPD), cardiovascular pathologies like myocardial infarction and hypertension, and neurological impairments, including cognitive decline and neurodegenerative disorders. Furthermore, such exposure contributes to reproductive and developmental toxicities, notably birth defects and low birth weight [32].

The epidemiological evidence reported in airport workers, including increased prevalence of respiratory symptoms in exposed occupational groups, supports the hypothesis that prolonged exposure to airport-related pollutants may contribute to adverse health effects. However, differences in exposure assessment methodologies and investigated populations highlight the need for standardized approaches in future occupational studies.

In detail, oxidative stress, inflammation, autophagy, and apoptosis are considered the primary pathways that mediate the detrimental effects of PM. Furthermore, indoor exposure to combustion-derived nanoparticles has been shown to induce epigenetic alterations in gene expression pathways related to xenobiotic metabolism, specifically through AhR-mediated upregulation of CYP1A1 and CYP1B1, independently of changes in DNA methylation, suggesting that alternative epigenetic mechanisms such as histone modifications or non-coding RNAs may be at play [1]. This finding is particularly relevant in airport occupational settings, where workers are chronically exposed to combustion aerosols from jet engines. The integration of biomarker-based approaches with environmental monitoring may provide additional information on early biological responses to occupational exposure. Biomarkers related to oxidative stress, inflammation, and xenobiotic metabolism could complement traditional exposure assessment methods and improve the characterization of exposure–response relationships among airport workers.

However, recent studies have identified several emerging mechanisms involved in PM-induced toxicity, including pyroptosis, ferroptosis, and epigenetic modifications. Additionally, xenobiotic toxicants can amplify the inflammatory and oxidative effects of PM. This amplification triggers a cascade ranging from immunotoxicity and mitochondrial dysfunction to genotoxicity, organ damage, and oncogenesis. Ultimately, these molecular disruptions contribute to elevated morbidity and mortality rates, particularly among vulnerable populations such as children, the elderly, and individuals with pre-existing pathophysiological conditions.

**Figure 4:**
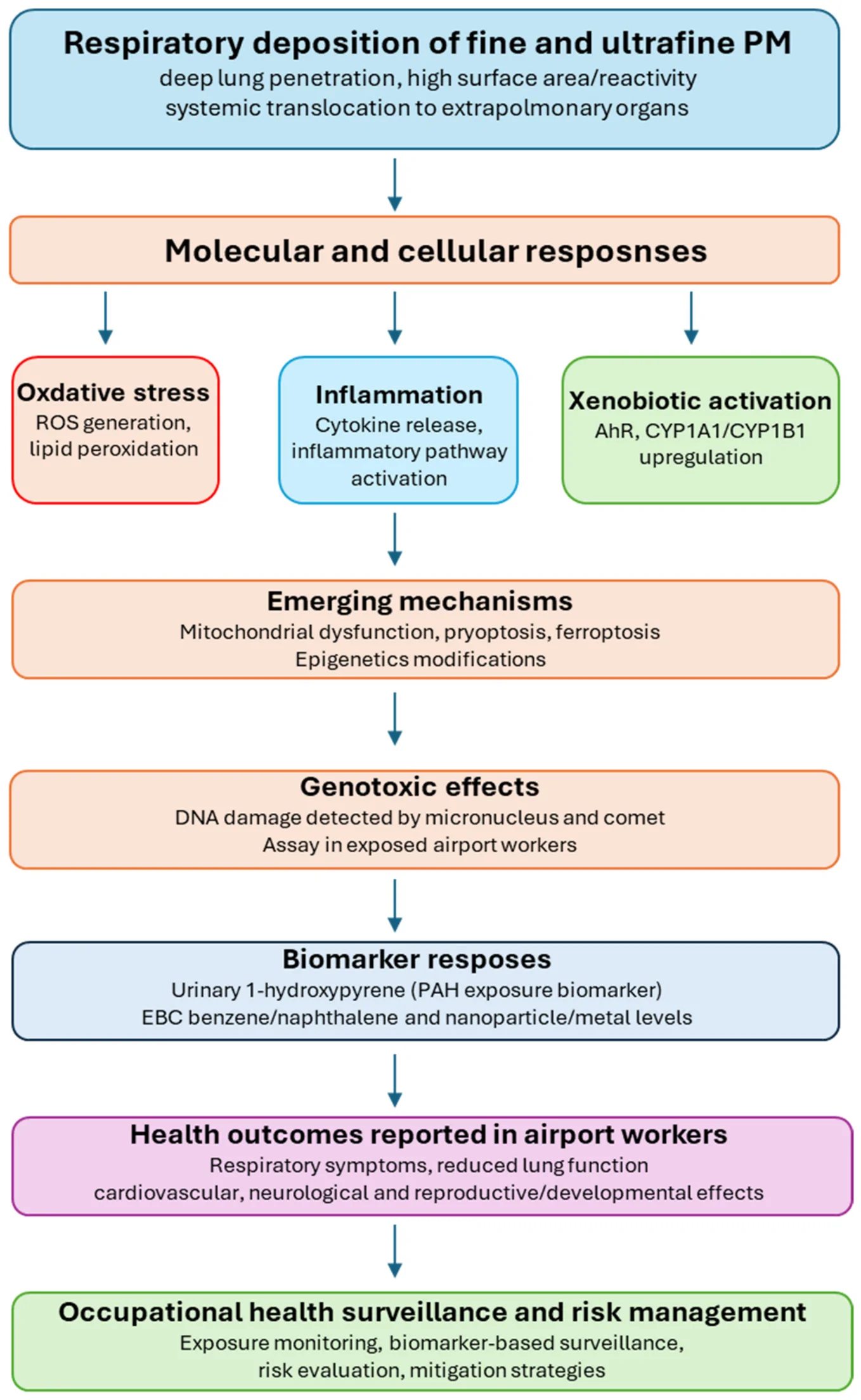
Mechanistic pathway linking inhalation of fine and ultrafine particulate matter to oxidative stress, inflammation, xenobiotic responses, emerging cellular mechanisms, and systemic health effects.

Figure 4 summarizes these biological pathways, from particle deposition to oxidative stress, inflammation, and xenobiotic pathway activation, through to the emerging mechanisms and systemic health effects discussed above.

Recent mitigation strategies in aviation include the development of sustainable aviation fuels (SAFs), such as hydroprocessed ester and fatty acid (HEFA)-based fuels. Although SAFs may contribute to reducing lifecycle greenhouse gas emissions and some combustion-related pollutants, their effects on particulate emissions require careful evaluation. Recent evidence indicates that SAF use may reduce elemental carbon and certain volatile organic compounds, while ultrafine particle concentrations may not decrease proportionally and could vary depending on fuel composition and engine operating conditions [17]. Therefore, both climate-related and human health endpoints should be considered when evaluating alternative aviation fuels.

This mini-narrative review has some limitations that should be considered when interpreting the findings. The available literature is characterized by heterogeneity in terms of study design, airport environments investigated, exposure assessment methodologies, pollutant metrics, and health outcomes evaluated. These differences limit the direct comparison of studies and prevent quantitative synthesis of the available evidence. Furthermore, although a structured literature search strategy was applied, the narrative nature of this review may not capture all available evidence. Future studies integrating standardized environmental monitoring, personal exposure assessment, and biomarker-based approaches are needed to better characterize exposure–response relationships among airport workers.

## 5. Conclusions

Overall, the evidence summarized in this mini-review indicates that occupational exposure among airport workers results from the interplay of aircraft- and ground-related combustion sources with indoor microenvironmental dynamics (Section 3.1 and Section 3.5), and that this exposure can be linked to oxidative stress, inflammatory responses, and xenobiotic pathway activation through biomarker-based studies (Section 4, Figure 3). A multidisciplinary approach integrating aerosol dynamics, indoor/outdoor exchange processes, and pathway-specific biological effects is needed to obtain a comprehensive mechanistic understanding of these exposures, together with their associated clinical and economic burden on occupational health systems.

Building on this evidence base, we propose that future risk assessment frameworks should combine source-specific toxicity data, biomarker-based surveillance, and systemic risk modelling. In this respect, emerging computational approaches, including AI-based exposure modelling and other advanced predictive tools, represent a promising but still largely unvalidated direction that was not directly assessed within the studies reviewed here; their integration into occupational exposure assessment should therefore be regarded as a priority for future research rather than as an established practice. Likewise, omics technologies such as genomics, proteomics, and metabolomics may offer additional insight into the molecular mechanisms underlying PM-induced health effects, although their usefulness in this specific occupational setting remains to be demonstrated.

Technological advancements in emission control, including improved filtration systems, cleaner combustion technologies such as sustainable aviation fuels, and optimized ventilation strategies, represent more immediately applicable approaches for reducing occupational exposure to airport-related pollutants. Consequently, we underscore the importance of integrated indoor/outdoor air quality monitoring, combined with biomarker-based surveillance, to support timely and evidence-based protection of airport workers while advanced predictive and omics-based tools are further developed and validated.

## Figures and Tables

**Table 1:** Overview of literature databases and structured search strategy.

Database	Keywords	Period Covered	Records Retrieved
PubMed	airport workers AND indoor air pollution	Jan 1996–May 2026	*n* = 23
Scopus	airport personnel AND indoor air quality	Jan 2005–May 2026	*n* = 7
Web of Science	occupational exposure AND indoor air evaluation	Jan 2012–May 2026	*n* = 2

**Table 2:** Main sources and formation/transfer mechanisms of airborne pollutants relevant to airport occupational exposure.

Source/Transfer Mechanism	Pollutant	Primary Combustion Emission; Near-Field Dispersion
Ground support equipment (diesel)	UFPs, black carbon, NOx, VOCs	Primary combustion emission at apron level
Road traffic (landside)	UFPs, PAHs, NOx, trace metals	Primary combustion emission; background contribution
Atmospheric processing	Secondary aerosol, semi-volatile organics	Photochemical reactions; condensation/partitioning modulated by temperature and humidity
Outdoor-to-indoor transfer	Fine and ultrafine PM of outdoor origin	Infiltration via HVAC systems, door/window opening; governed by I/O ratio and filtration efficiency
Indoor sources	Resuspended particles, VOCs	HVAC operation, building materials, indoor activities

**Table 3:** Prevalences and Odds Ratios (ORs) of respiratory symptoms.

Symptom	Exposed %	Controls %	Crude OR	Adjusted ORs (95% CI)
Cough	15.1	6.6	2.53	3.41 (1.26–9.28)
Dyspnea	22.6	13.8	2.06	2.34 (1.05–5.18)

95% CI: 95% confidence interval. Odds ratios adjusted for age (<40 or ≥40 yr), education (<high school or ≥high school), marriage (yes or no), duration of employment (<10 or ≥10 yr), smoking status (current smokers or ever/never smokers), and previous occupational dust or fume exposures (yes or no). Significant at *p* < 0.05.

**Table 4:** Job exposure groups.

Airport Employees	Job Transport	Job Location	Level of Exposure
Baggage handlers/aircraft mechanics	Push back tractor	Apron	High
Catering drivers	Diesel-powered high loader	Apron	Medium
Cleaning staff	Diesel-powered high loader or lorry	Apron	Medium
Airside security		Apron	Medium
Landside security/office staff		Airside	Low

## Data Availability

No new data were created or analyzed in this study. Data sharing is not applicable to this article.

## Abbreviations

UFPs	ultrafine particles
PM	particular matter
BC	black carbon
PAHs	polycyclic aromatic hydrocarbons
WHO	World Health Organization
OPEs	organophosphate esters
GC–MS	gas chromatography–mass spectrometry
EBC	exhaled breath condensate
HVAC	heating, ventilation, and air-conditioning
ICAO	International Civil Aviation Organization
COPD	chronic obstructive pulmonary disease
AI	artificial intelligence
SAFs	sustainable aviation fuels
HEFA	hydroprocessed fatty acid ester
